# Myiasis-induced sepsis: a rare case report of *Wohlfahrtiimonas chitiniclastica* and *Ignatzschineria indica* bacteremia in the continental United States

**DOI:** 10.1097/MD.0000000000013627

**Published:** 2018-12-28

**Authors:** Travis B. Lysaght, Meghan E. Wooster, Peter C. Jenkins, Leonidas G. Koniaris

**Affiliations:** a Trauma and Acute Care Surgery, OhioHealth Grant Medical Center, Columbus, Ohio; b General Surgery, OhioHealth Doctors Hospital, Lincoln Village, Columbus; c Trauma and Acute Care Surgery, Indiana University Health Methodist Hospital, Indianapolis, Indiana.

**Keywords:** bacteremia, green bottle fly, *Ignatzschineria indica*, *Lucilia sericata*, sepsis, treatment of cavitary and cutaneous myiasis, *Wohlfahrtiimonas chitiniclastica*

## Abstract

**Rationale:**

The presentation of sepsis and bacteremia in cutaneous and cavitary myiasis is uncommon. We present a patient, residing in a temperate region of the United States, with myiasis and sepsis from the emerging human pathogens *Wohlfahrtiimonas chitiniclastica* and *Ignatzschineria indica*.

**Patient concerns:**

A 37-year-old male patient with an 8-month history of chronic lymphedema and ulcers of the lower left extremity presented with myiasis of the left foot and leg. The patient was initially seen by his family practitioner many times and was prescribed antibiotics which he could not afford. Debridement of the myiasis was not conducted by the family practitioner due to the belief that the patient's current state of myiasis would effectively debride and eventually heal the chronic ulcers along with multiple antibiotic regimens. Over the 8-month period, the patient developed a progressive, painful, necrotizing infection of his lower left extremity.

**Diagnoses:**

Physical examination clearly showed myiasis of the patient's lower left extremity, believed to be caused by *Lucilia sericata* (green bottle fly). Blood cultures revealed the presence of *Providencia stuartii*, *W chitiniclastica*, and *I indica* to be the underlying cause of sepsis and bacteremia.

**Interventions:**

All visible maggots were extracted, debridement of devitalized tissue was performed, and the leg ulcers were wrapped in pH neutral bleach. The patient was initially treated with a broad-spectrum antibiotic regimen of vancomycin, clindamycin, piperacillin, and tazobactam which, following clinical improvement, was de-escalated to cefepime.

**Outcomes:**

The fly larvae and maggots were removed from the extremity by scrubbing, pulse lavage, and filing away the callused tissue. Additionally, the patient's sepsis and bacteremia, caused by *W chitiniclastica* and *I indica*, were successfully treated through antibiotic intervention. Amputation was avoided.

**Lessons:**

The use of pulse lavage and chlorhexidine-soaked brushes for the removal of cavitary myiasis is an effective and minimally invasive procedure which does not cause additional damage to surrounding tissue. *W chitiniclastica* and *I indica* are emerging bacteria that have known association to parasitic fly myiasis in humans and are capable of causing sepsis and/or bacteremia if not accurately identified and treated promptly.

## 1 Introduction

Myiasis is an affliction of parasitic flies in the larvae stage which infest human or other vertebrates’ necrotic or, in some cases, living tissue. Myiasis is broken into categories regarding the location of infestation, but the two main categories of myiasis are cutaneous and cavitary.^[1]^ Myiasis most often occurs with patients who have unhealed, open wounds which allow for parasitic flies to leave their larvae.^[2]^ While there are clinical applications to the use of controlled myiasis for debridement of necrotic tissue, there are also cases where accidental myiasis occurs.^[1,3]^ Typically, people of low economic means and those who have poor hygiene have increased susceptibility—these populations include, but are not limited to, homeless, alcoholics, low-income farmers, and those who lack needed assisted care (i.e., elderly, paraplegics, etc.).^[1,3–8]^ Due to higher likelihood of parasitic flies in their area of residence, those who live in close proximity to livestock are at increased risk of myiasis as well.^[8]^ In the past, myiasis was thought to be almost exclusively associated with tropical and subtropical regions, but in recent years, more literature has begun to surface about cases in temperate climates.^[1,3]^ As with any sort of infestation of parasites, myiasis causes concerns for the possibility of secondary bacterial infection, since certain species of fly and their larvae harbor associated bacteria. For this reason, myiasis should prompt healthcare providers to suspect possible bacterial infection, both of the tissue and the blood.^[7,9]^

Of particular concern in our case, *Wohlfahrtiimonas chitiniclastica* and *Ignatzschineria indica* are bacteria which have been known to be associated with myiasis. *W chitiniclastica* and *I indica* are aerobic, gram-negative rods that are nonmotile and nonspore-forming.^[10,11]^ Both of these bacteria are unusual human pathogens, but they have been documented in relation to maggot infestations in the last few years.^[2,4–7,9,11–15]^ Just as myiasis may indicate an underlying bacterial infection, the presence of bacteremia with either *W chitiniclastica* or *I indica* in humans with no observable larvae may indicate occult myiasis.^[6]^

Currently, there is a paucity of literature about the subject of myiasis and even less literature on *W chitiniclastica* and *I indica* bacteremia and/or sepsis. Concerning sepsis and/or bacteremia induced by myiasis, there are only a handful of cases in existence worldwide with direct links.^[2,4,7,11,14,15]^ This case of myiasis is the first presented in literature where a patient experiences coinfection of *W chitiniclastica* and *I indica*, causing bacteremia and sepsis.

## 2 Case presentation

A 37-year-old male patient was transferred from an outside emergency department with concern of self-described “trench foot” that he reported having for 8 months. The patient worked as a “scrapper” in the local area, wading into swamps and ditches to retrieve junk metal. The patient reported that his feet were often wet, due to his job. When he noticed chronic, progressive, painful, necrotizing infection of his lower left extremity, he sought medical attention. On various occasions during the 8-month time period, he had been prescribed antibiotics, but he did not follow through with them due to financial limitations. The patient's lower left extremity first developed myiasis and ulceration of the toes and lateral calf.

**Figure 1:**
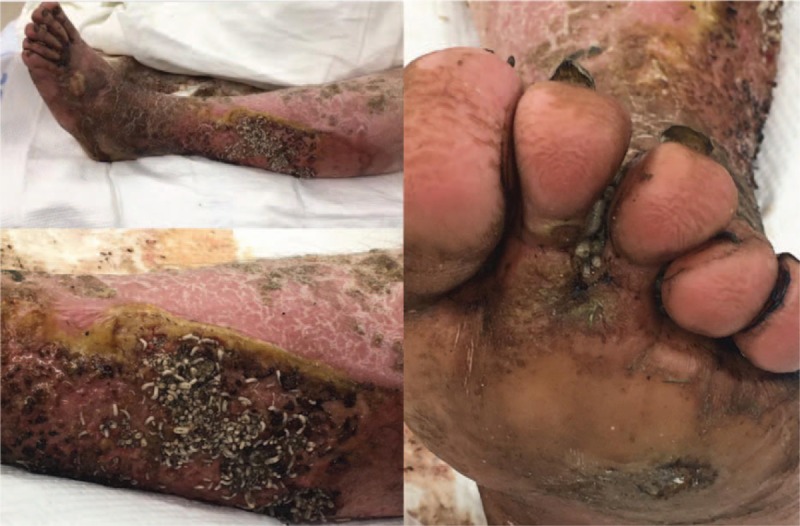
Pre-operative photos (HD#0) of the patient's lower left extremity, presenting with cutaneous and cavitary myiasis and necrotizing infection. Lower left-hand photo shows the patient's left lateral calf, and the right-hand photo shows the patient's left foot. The upper left-hand photo depicts the lower left extremity from below the knee.

Upon admission, the patient described fevers and chills for several weeks prior. His vitals showed a body temperature of 37.2°C, heart rate of 122 beats per minute, blood pressure of 114/71 mm Hg, respiratory rate of 16 breaths per minute, and 95% oxygen saturation on room air. The patient's lower left extremity presented myiasis between the toes and on the lateral left calf with multiple cavitary lesions and diffused areas of callus, significant erythema, and edema (Fig. 1). The myiasis was believed to be caused by *Lucilia sericata*, more commonly known as the green bottle fly. *Lucilia sericata* was suspected due to the appearance of the larvae, the vast presence of the species in the United States, including temperate regions, and their association to similar cases of myiasis in literature.^[2]^ The patient's medical history revealed spina bifida, tobacco use, poor dentition, and a previous burn with “molten steel” requiring a skin graft on the right leg. The patient was resuscitated, blood cultures were drawn, and he received vancomycin (1.75 g, every 12 h), clindamycin (600 mg, every 6 h), and a combination of piperacillin and tazobactam (4.5 g, every 8 h).

**Figure 2:**
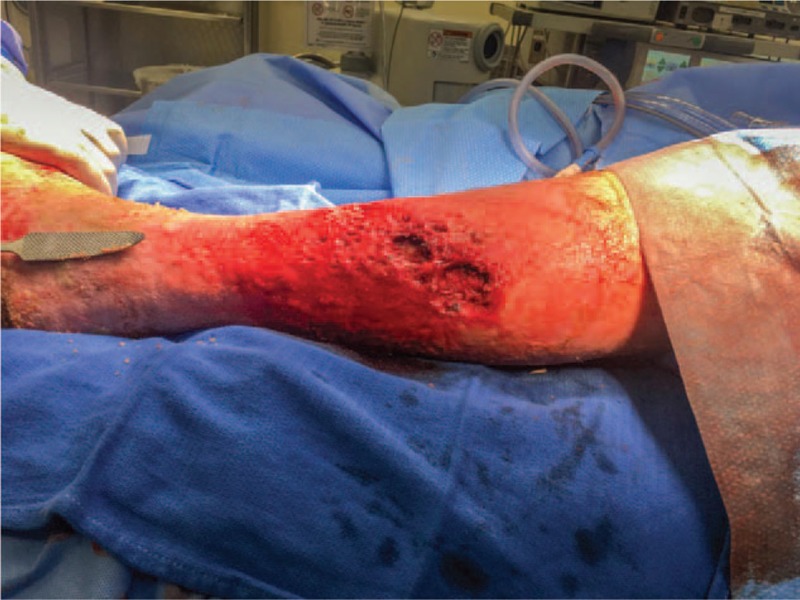
Intraoperative photo (HD#0) of the patient's left leg just after clearing of the cutaneous and cavitary myiasis.

The patient's maggot infestation was removed mechanically in the operating room on hospital day zero (HD#0). Operative goals included the need to reduce contamination and salvage as much viable skin as possible on the patient's leg. Cutaneous maggots were removed by manually scrubbing with chlorhexidine-soaked brushes along with removal of maggots with forceps when necessary. Pulse lavage, using a closed-system to prevent unnecessary contamination, was used to extract cavitary maggots and irrigate the cavitary lesions.^[16]^ Chlorhexidine-soaked brushes were also used for cavitary maggots when applicable. A surgical file was used to break up calluses along the wounds (Fig. 2).

Given the high likelihood of multiple pathogens in the large wound area, the wound was packed with buffer-neutral bleach which had broad spectrum antibacterial and fungal activity. The patient tolerated the initial therapy well, with no notable remaining maggots at the first dressing change (12 h post-operation) and continued on his antibiotic regimen.

**Figure 3:**
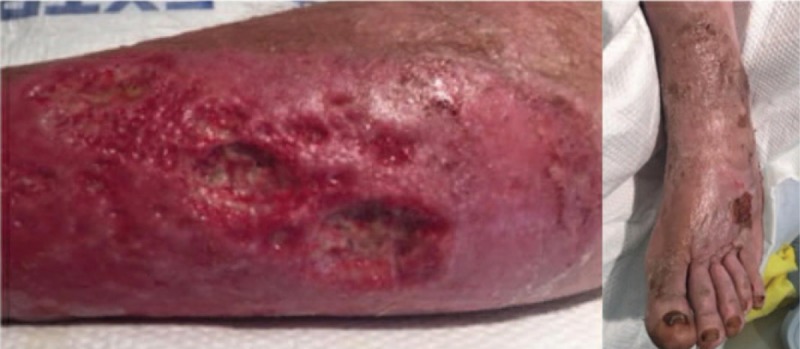
Post-operative photo (HD#6) of the patient's left lateral calf (left-hand photo) and left foot (right-hand photo).

A post-operative MRI (POD#1) of the patient's leg revealed no evidence of osteomyelitis in the tibia, fibula, or ankle. Wounds were cared for with daily pulse lavage, sharp selective debridement, and dressed with Acticoat (Smith & Nephew, London, UK). The leg edema was treated with compression and elevation. Initial blood cultures grew *W chitiniclastica*, *I indica*, and *Providencia stuartii*. The patient responded well to the 3-day course of antibiotics and was de-escalated to cefepime (2 g, every 8 h) on HD#2. Subsequent blood cultures were negative for any growth. On HD#2, a transthoracic echocardiogram was performed to assess for endocarditis and was unremarkable. On HD#4, there was increased granulation tissue with decreasing erythema and edema; therefore, the wound was dressed with Hydrofera Blue (Advanced Tissue, Little Rock, AR) and a light Coban self-adherent wrap (3M, St. Paul, MN) for compression (Fig. 3). Upon discharge on HD#10, cefepime was discontinued and current dressing changes were continued with follow-up in outpatient wound care clinic. The patient was recommended to discontinue prolonged exposure to water to decrease the likelihood of recurrence. At 6 months, the patient's wounds were healed, no residual pain or edema was present, and his left leg was fully ambulatory and functional.

The institutional review board of Indiana University Health Methodist Hospital waved the need for approval since the patient was treated by physicians who are reporting this case and personal health information was de-identified, per Indiana University Standard Operating Procedures for Research Involving Human Subjects: Section 3.2.10.3. The patient gave informed consent, allowing this case report.

## 3 Discussion

Accidental myiasis inducing sepsis and/or bacteremia is a potentially lethal clinical entity. There are currently limited publications regarding the connection of specific species of larvae to strains of bacteria and the successful management of their infestations and infections.^[9,17]^

This case study is notable for the method in which the myiasis was removed. Up to now, literature for cutaneous myiasis suggests that debridement be done manually using forceps and Vaseline (Unilever, Rotterdam, NL), or Vaseline-like substances, to suffocate the larvae, driving them from deeper tissue up to the surface for easier removal.^[18]^ For cavitary myiasis, literature suggests the surgical debridement of the maggots using incisions and, if necessary, the resection of infected tissue.^[1]^ This study presents an alternative method for larvae removal that is minimally invasive—the use of pulse lavage and chlorhexidine-soaked brushes. In addition to forceps being used for removal of cutaneous larvae, pulse lavage and chlorhexidine-soaked brushes are capable of clearing an infested cavitary area while not causing additional destruction of viable tissue.

This case also underscores that myiasis frequently does not require amputation. In this case, the pulse lavage and chlorhexidine-soaked brushes allowed for this goal to be achieved and for the patient's leg to be preserved with a near-intact functional result.

Another educational point of this case is that community-acquired myiasis infections can result in serious systemic bacterial infections. Although blow flies are commonly used in maggot therapy, they are not the same as the flies found in the environment. Blow flies and green bottle flies found in the environment may harbor bacteria that are not found in medical-grade maggots. Medical-grade maggots are free of pathologic bacteria because they are grown in a sterile, cultured environment and are washed with dilute disinfectant before being packaged in sterile containers.^[19]^ When used, multiple applications may be required depending on the size and the amount of necrotic tissue.^[20]^ Thus, in incidents of accidental cutaneous or cavitary myiasis, antibiotics alone may be insufficient, and therapy directed to remove the myiasis should be considered. Treatment objectives should include manual debridement of myiasis, proper wound care, systemic antibiotic treatment, and ongoing wound hygiene to promote wound healing and prevent the infestation from progressing.^[1]^

Finally, this report presents a patient with sepsis and bacteremia caused by *W chitiniclastica* and *I indica* bacteria which was spread by parasitic fly larvae believed to be *Lucilia sericata* (green bottle fly). Both of these bacteria are rare, and myiasis cases where sepsis and/or bacteremia was caused by either *W chitiniclastica* or *I indica* have limited literature.^[2,4,7,11,14,15]^ This case of myiasis presents the first patient in literature to experience coinfection of *W chitiniclastica* and *I indica*, causing bacteremia and sepsis (Table 1). This confirms that *W chitiniclastica* and *I indica* are emerging bacteria and that myiasis of parasitic flies, even in temperate regions, can spread such bacterial infections.

**Table 1:**
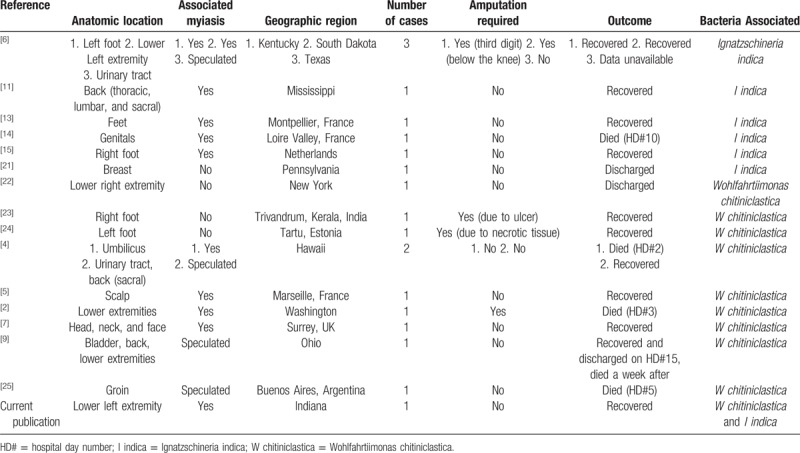
Current literature review for human cases of *Wohlfahrtiimonas chitiniclastica* and *Ignatzschineria indica.*

## Author contributions

**Conceptualization:** Leonidas G. Koniaris.

**Supervision:** Peter C. Jenkins, Leonidas G. Koniaris.

**Writing – original draft:** Meghan E. Wooster, Travis B. Lysaght.

**Writing – review & editing:** Travis B. Lysaght, Meghan E. Wooster, Peter C. Jenkins, Leonidas G. Koniaris.
